# Cardiovascular disease and resuscitated septic shock lead to the downregulation of the H_2_S-producing enzyme cystathionine-γ-lyase in the porcine coronary artery

**DOI:** 10.1186/s40635-017-0131-8

**Published:** 2017-03-21

**Authors:** Tamara Merz, Tatjana Stenzel, Benedikt Nußbaum, Martin Wepler, Csaba Szabo, Rui Wang, Peter Radermacher, Oscar McCook

**Affiliations:** 1 Universitätsklinik Ulm, Institut für Anästhesiologische Pathophysiologie und Verfahrensentwicklung, Helmholtzstrasse 8, 89081 Ulm, Germany; 2 Universitätsklinik Ulm, Klinik für Anästhesiologie, Albert-Einstein-Allee 23, 89081 Ulm, Germany; 3 Department of Anesthesiology, The University of Texas Medical Branch at Galveston, 601 Harborside Drive, Galveston, TX 77555 USA; 4 Department of Biology, Laurentian University, Sudbury, ON Canada; 5 Institute of Anesthesiological Pathophysiology and Process Engineering, University Medical School, Helmholtzstrasse 8-1, 89081 Ulm, Germany

**Keywords:** Endothelial NO synthase, Oxidative stress, Shock, Peroxynitrite, Blood vessels

## Abstract

**Background:**

Downregulation of the hydrogen sulfide (H_2_S)-producing enzymes cystathionine-γ-lyase (CSE), cystathionine-β-synthase (CBS), and/or 3-mercaptopyruvate sulfurtransferase (3-MST) is associated with chronic cardiovascular pathologies. Nevertheless, equivocal data are available on both the expression and function of these enzymes in coronary arteries (CA). We recently reported that atherosclerotic pigs subjected to sepsis developed impaired cardiac function, which coincided with decreased myocardial CSE expression and increased nitrotyrosine formation. To define the endogenous source(s) of H_2_S in the CA, we studied the expression of CBS, CSE, or 3-MST in the CA of pigs subjected to septic shock with/without pre-existing cardiovascular co-morbidity.

**Methods:**

Anesthetized and instrumented FBM "familial hypercholesterolemia Bretoncelles Meishan" pigs with high-fat diet-induced hypercholesterolemia and atherosclerosis were subjected to polymicrobial septic shock, or sham procedure, and subsequent intensive care therapy for 24 h. Young German domestic pigs were used as naïve controls. CSE, CBS, 3-MST, HO-1, eNOS, and nitrotyrosine expression was quantified by immunohistochemistry of formalin-fixed paraffin sections.

**Results:**

FBM pigs, in the absence of septic shock, showed decreased CSE expression in the media. This decrease became more pronounced after sepsis. The expression pattern of HO-1 resembled the pattern of CSE expression. CBS protein was not detected in the media of any of the CA examined but was localized to the adventitia and only in the atheromatous plaques containing foam cells of the CA, in regions that also displayed abundant nitrotyrosine formation. The CBS expression in the adventitia was not associated with nitrotyrosine formation. 3-MST expression was not found in any of the CA samples.

**Conclusions:**

We hypothesize that (i) the reduced CSE expression in FBM pigs may contribute to their cardiovascular disease phenotype and moreover (ii) the further decrease in CA CSE expression in sepsis may contribute to the sepsis-associated cardiac dysfunction.

## Background

The endogenous gasotransmitter hydrogen sulfide (H_2_S) has been shown to play a pivotal regulatory role in the cardiovascular system, where it contributes to the maintenance of basal vasorelaxant tone and serves as an endogenous stimulator of angiogenesis [1]. Cystathionine-γ-lyase (CSE) is considered one of the key H_2_S-producing enzymes in the cardiovascular system. Downregulation of CSE is associated with chronic cardiovascular pathology, e.g., hypertension and atherosclerosis [2, 3]. Nevertheless, equivocal data are available on both the expression and the function of these endogenous enzymes in coronary arteries (CA). On the one hand, Cheang (rat, ex vivo CA, KCl vasodilation), Casalini (dogs, coronary microvasculature), and Kuo (mouse, rat CA, and human endothelial cells) reported little to no CSE contribution; on the other hand, Chai (mouse, ex vivo CA) and Hedegaard (porcine CA, ex vivo de-endothelialized) have found that CSE was involved in CA physiology [4–8]. The discordant data may be reflective of the differing experimental designs (pharmacological and genetic inhibition, intact or de-endothelialized ex vivo isolated coronary arteries or microvasculature, in vitro cell culture) and the possible role of species differences, as well as differences in the strains, gender, and age of the animals studied [9].

Most studies focusing on pathophysiology utilize young animals, without any cardiovascular disease background, even though these animals are clearly not reflective of the pathophysiology of the patient populations. Particularly, the role of atherosclerosis (a common underlying disease to many acute diseases in humans) is not modeled by the use of these naive/young animal models. In atherosclerosis (mainly affecting large- and medium-sized vessels and characterized by plaque formation, the presence of foam cells, stiffening of the vasculature, and decreased flow), CSE has been shown to play an important role [10]. Both genetic and pharmacological inhibition of CSE was found to lead to accelerated atherosclerosis development, while administration of H_2_S suppressed the development of atherosclerosis [10–13].

Septic patients with CA disease (CAD) present with an impaired myocardial compliance in comparison to patients without CAD [14]. Moreover, we recently reported that septic pigs on the background of cardiovascular disease exhibit increased nitrotyrosine formation and significantly decreased CSE expression in the myocardium, which coincided with an impairment of their cardiac function [15].

In general, pigs have been shown to be closer to humans than mice in more than 80% of the immune parameters examined [16]. Regarding the expression of the endogenous H_2_S enzymes, the pig has also been shown to be closer to the human than the mouse [3, 17]. In order to better understand the endogenous source for H_2_S synthesis in the CA under normal and pathologic conditions, we used both young healthy German domestic pigs (YGP) and swine with a naturally occurring mutation homozygous for the R84C low-density lipoprotein (LDL) gene, “familial hypercholesterolemia Bretoncelles Meishan” (FBM), fed an atherogenic diet. FBM animals are known to exhibit significantly higher cholesterol levels compared to healthy swine of the same age [18–20], which produces the biomarker pattern of hypercholesterolemia, increased oxidative stress, reduced creatinine clearance, and lower blood levels of nitric oxide (NO) metabolites [20] reminiscent of the biomarker pattern found in patients with hypercholesterolemia-induced atherosclerosis [21]. In the present study, we performed a post hoc analysis of CA collected in a previous study [22] to investigate the endogenous expression of the H_2_S enzymes in porcine CA. We have compared the expression patterns of young naïve pigs, pigs with pre-existing cardiovascular co-morbidity, and pigs with pre-existing cardiovascular co-morbidity subjected to septic shock.

## Methods

The study was approved by the University of Ulm Animal Care Committee and the Federal Authorities for Animal Research. The experiments were performed in adherence to the National Institute of Health Guidelines on the Use of Laboratory Animals and the European Union “Directive 2010/63/EU on the protection of animals used for scientific purposes.” This is a post hoc study performed on available tissue from the vehicle-treated group of a previous study [22] and unpublished sham-operated animals studied in the same time period. Briefly, male castrated FBM (age 15–30 months, 69 kg (65–73 kg)) with a high-fat diet-induced hypercholesterolemia and atherosclerosis [19] underwent polymicrobial septic shock (*n* = 8) induced by inoculation of autologous feces into the abdominal cavity, or sham procedure, i.e., abdominal saline injection (*n* = 5), and subsequently received intensive care therapy for 24 h. Young German domestic pigs (YGP, *n* = 5) (age 3–6 months, 67 kg (57–72 kg)), kept under the same breeding conditions as the FBM pigs and fed a standard diet (Bonimal SK Mast 130S, BayWa), were anesthetized before sacrificing without further instrumentation and used as naïve controls. Anesthesia and surgical instrumentation have been previously described in detail [22, 23]. In brief, for all animals, anesthesia was induced with propofol and ketamine to allow endotracheal intubation and was maintained thereafter with continuous i.v. pentobarbitone and pancuronium and intermittent buprenorphine. Ventilator settings were fraction of inspired O_2_ (FiO_2_) 0.35, positive end expiratory pressure (PEEP) 10 cmH_2_O, tidal volume 8 ml/kg, respiratory rate 10 to 12 breaths/min adjusted to maintain arterial PCO_2_ = 35 to 40 mmHg, inspiratory (*I*)/expiratory (*E*) ratio 1:1.5, peak airway pressure <40 cmH_2_O, and modified to *I*/*E* ratio 1:1 and PEEP 12 or 15 cmH_2_O if the ratio of arterial O_2_ partial pressure (PaO_2_)/FiO_2_ is <300 or <200 mmHg, respectively [22]. The septic and sham pigs had the right jugular vein and carotid artery exposed for the insertion of a central venous catheter sheath and the placement of a balloon-tipped pulmonary artery catheter to measure central venous pressure (CVP) and a thermistor-tipped arterial catheter for blood pressure (mean arterial pressure, MAP) recording and transpulmonary single indicator thermodilution–cardiac output measurement. Ringer’s solution was continuously infused as maintenance fluid (10 ml/(kg h)). As needed, animals received hydroxyethyl starch to maintain cardiac filling pressures during surgery.

### Experimental protocol

Atherogenic diet (1 kg daily, 1.5% cholesterol, 20% bacon fat) was fed for at least 9 months prior to the experiments. Post anesthesia and surgical instrumentation, the supernatant (3 ml/kg) of 1.0 g/kg autologous feces incubated in 500 ml 0.9% saline for 12 h at 38 °C, or saline only as sham procedure, was injected into the abdominal cavity via the abdominal drainage tubes. Hydroxyethyl starch (10 ml/(kg h)), 5 ml/(kg h) if CVP or PAOP is >18 mmHg, allowed maintaining hyperdynamic hemodynamics. If necessary, norepinephrine was infused and titrated to maintain MAP at baseline values (discontinued if the heart rate was ≥160 beats/min to avoid tachycardia-induced myocardial ischemia) [22]. Twenty-four hours after induction of fecal peritonitis, animals were sacrificed with KCl after further deepening of anesthesia.

### Immunohistochemistry

CA were dissected immediately postmortem and then fixed in formalin (fixation identical for all samples), dehydrated, and embedded in paraffin blocks. Paraffin sections (3–5 μm) were cut, deparaffinized in xylene, and rehydrated with a graded series of ethanol to deionized water. After heat-induced antigen retrieval, the slides were blocked with 10% normal sera (Jackson ImmunoResearch) before incubating in primary antibody (1° ab, anti-nitrotyrosine (Millipore)) and CSE: anti-CTH (Abnova), anti-CBS (Santa Cruz Biotechnology), anti-MPST (Sigma), anti-adipophilin (Progen), anti-eNOS (BD), and anti-HO-1 (Abnova). Primary antibody detection was performed by alkaline phosphatase-conjugated secondary antibodies (anti-guinea pig, Jackson ImmunoResearch) or by Dako REAL detection system (anti-mouse, anti-rabbit) and visualized with red chromogen (Dako REAL; Dako) followed by counterstaining with hematoxylin (Sigma). Slides were visualized using a Zeiss Axio Imager A1 microscope with a ×10 objective. Quantification for intensity was performed using the AxioVision 4.8 software (Zeiss) [24]. Data are presented as densitometric sum red.

In particular for cystathionine-β-synthase (CBS) and 3-mercaptopyruvate sulfurtransferase (3-MST), where we found unusual or negative results, we include porcine-positive control tissue that is known to express both proteins. These control stains of pig adrenals clearly demonstrate the specificity of the 3-MST and CBS antibodies for porcine proteins (see Fig. 3e, f). The 3-MST antibody has previously been published in rodent coronary arteries [8]. The specificity of the antibody has additionally been confirmed in a blast search (88% identity of the immunogenic amino acid sequence to the pig protein vs. 81–82% for mouse and rat). The CBS antibody has been used on the pig and published previously [3].

### Statistical analysis

Data are presented as median (quartiles). After exclusion of normal distribution using the Kolmogorov–Smirnov test, differences between groups were analyzed with a one-way Kruskal–Wallis analysis of variance on ranks followed by a post hoc Dunn test. Inter-group differences were tested using a Mann–Whitney rank sum test.

## Results

Table 1 summarizes the physiological data (medians and interquartile range): Septic shock is defined as a state of acute circulatory failure with a need for vasopressor support to maintain MAP and elevated lactate (≥2 mmol/l) in spite of adequate fluid resuscitation [25]. The septic animals were characterized by decreased MAP (*p* = 0.017) despite supportive volume therapy and significantly higher continuous i.v. infusion of noradrenaline and also by significantly higher heart rate (*p* = 0.004). The target MAP could not be achieved without exceeding the maximally tolerated heart rate of 160 beats/min. Sepsis led to a significantly decreased PaO_2_ (*p* = 0.043), progressive lactic acidosis (*p* = 0.004), and more negative base excess (*p* = 0.004), all characteristic signs of septic shock.

**Table 1:** Physiological data

		Baseline	24 h
NoA (μg/(kg min))	Sham	0.06 (0.02; 0.13)	
	Sepsis	1.23 (0.66; 3.26)^a^	
Mean arterial pressure (mmHg)	Sham	100 (90; 106)	103 (94; 119)
	Sepsis	103 (91; 112)	65 (61; 81)^a, b^
Central venous pressure (mmHg)	Sham	8 (7; 13)	10 (9; 15)^b^
	Sepsis	10 (6; 13)	17 (14; 18)^b^
Cardiac output (ml/(kg min))	Sham	61 (52; 79)	64 (42; 92)
	Sepsis	64 (52; 69)	87 (62; 130)^b^
pO_2_ (mmHg)	Sham	158 (142; 180)	159 (138;177)
	Sepsis	170 (161; 183)	93 (62; 155)^a, b^
pCO_2_ (mmHg)	Sham	35 (35; 39)	35 (33; 36)
	Sepsis	38 (34; 40)	35 (32; 44)
arterial pH	Sham	7.46 (7.44; 7.46)	7.44 (7.43; 7.46)
	Sepsis	7.45 (7.43; 7.48)	7.37 (7.19; 7.43)^a, b^
Lactate (mmol/l)	Sham	1.4 (1.0; 1.6)	0.6 (0.6; 1.2)
	Sepsis	0.8 (0.6; 1.5)	6.1 (2.0; 10.7)^a, b^
Base excess (mmol/l)	Sham	1.1 (0.8; 1.8)	−0.1(−1.45; 0.65)
	Sepsis	1.5 (0.4; 2.3)	−8.5 (−14.6; −3.7)^a, b^

Physiological data, presented as median (interquartile range, IQR). Sham *n* = 5, *s*epsis *n* = 8

*NoA* norepinephrine infusion rate, *pO*_*2*_ partial oxygen pressure, *pCO*_*2*_ partial carbon dioxide pressure

^a^Significant compared to sham

^b^Significant compared to baseline

**Fig. 1:**
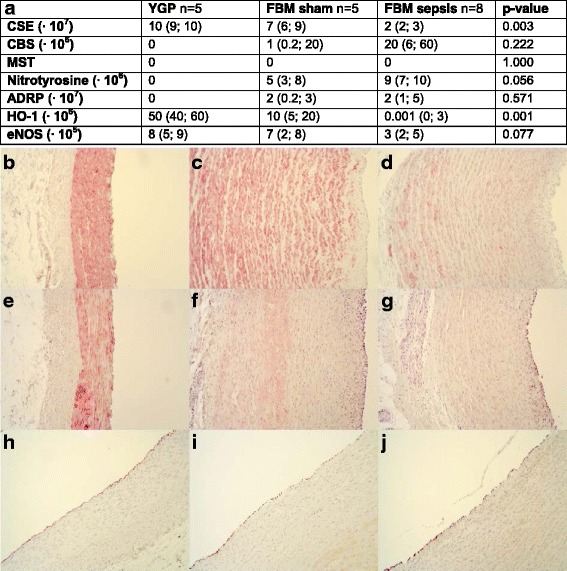
**a** Quantification of densitometric analysis of immunohistochemistry. Data are presented as median (IQR). Representative pictures ×10. **b** CSE YGP, **c** CSE FBM sham, **d** CSE FBM sepsis, **e** HO-1 YGP, **f** HO-1 FBM sham, and (**g**) HO-1 FBM sepsis. **b**–**g** show a cross-section of the vessel wall with the luminal side to the *right*. **h** eNOS YGP, **i** eNOS FBM sham, and (**j**) eNOS FBM sepsis. **h**–**j** show a cross-section of the vessel wall with the luminal side to the *upper left corner*

The YGP presented with high CSE expression, which was localized to the medial region of the CA. In contrast, pigs with ubiquitous atherosclerosis had 40% lower CSE expression in their CA. The reduced CSE expression was even more pronounced when pigs with CAD were subjected to sepsis (*p* = 0.003; Fig. 1). The expression pattern of heme oxygenase-1 (HO-1) resembled the expression pattern of CSE: basal levels of HO-1 expression were high in the YGP, were reduced with CAD, and were further attenuated with the combination of CAD and sepsis (*p* = 0.001; Fig. 1). The expression of endothelial nitric oxide synthase (eNOS) showed a tendency towards a reduction in the septic group (Fig. 1) which, however, did not reach statistical significance (*p* = 0.077).

**Fig. 2:**
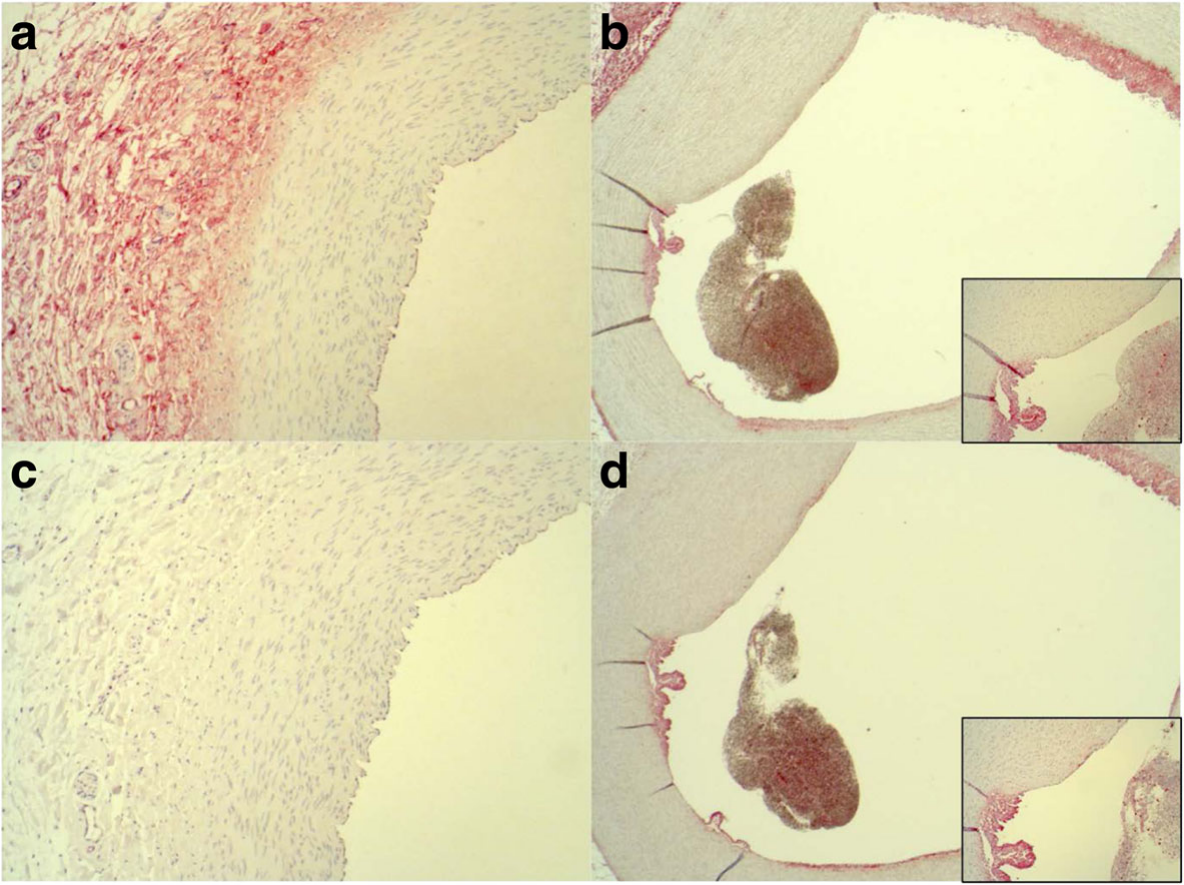
**a** CBS expression YGP 10×. **b** CBS expression FBM 2.5× (*inset* 10×). **c** Nitrotyrosine YGP 10×. **d** Nitrotyrosine FBM 2.5× (*inset* 10×). The pictures show cross-sections of coronary arteries; the vessel depicted in (**b**) and (**d**) contains a blood clot

**Fig. 3:**
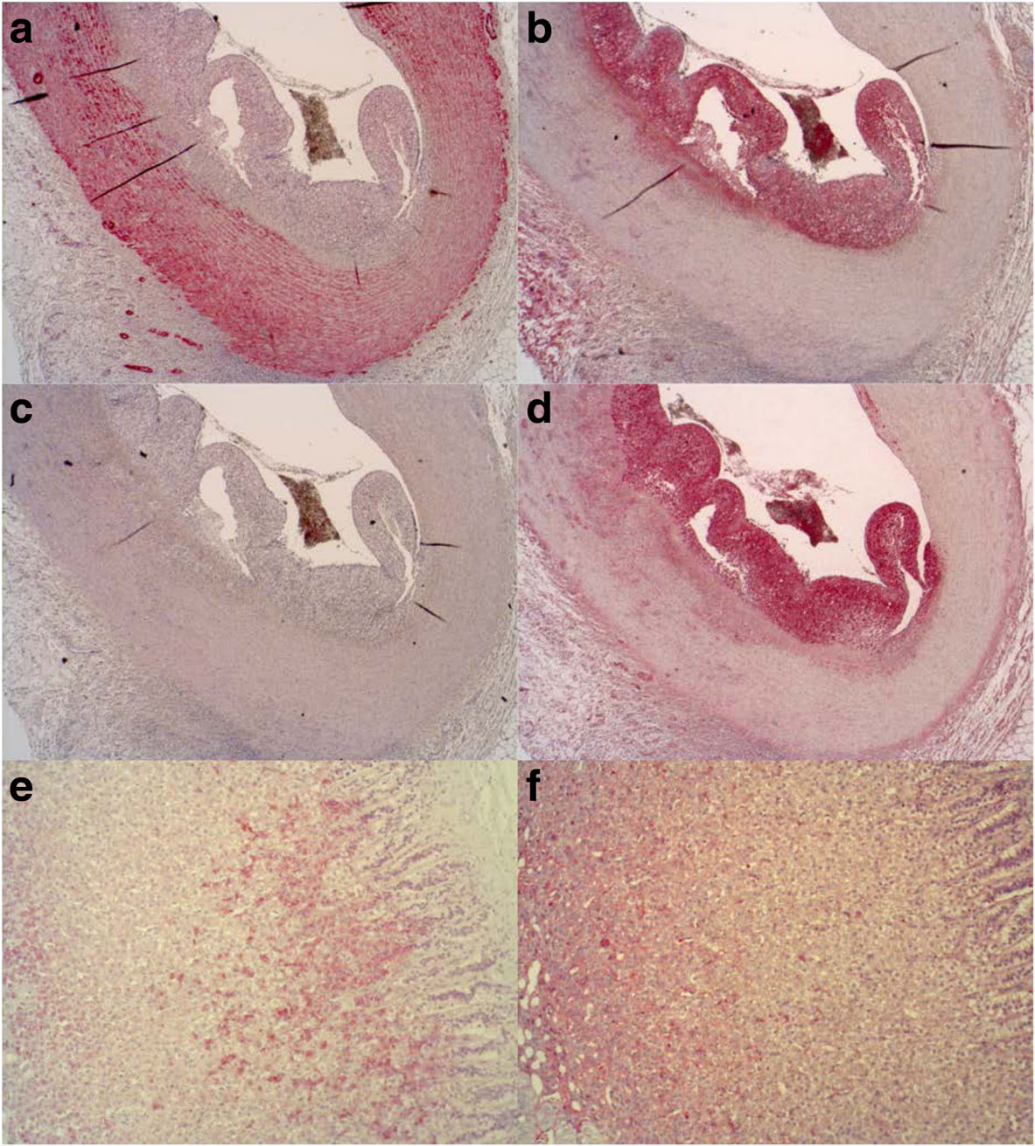
Protein expression in CAD. **a** CSE 2.5×. **b** CBS 2.5×. **c** MST 2.5×. **d** ADRP 2.5×. **a**–**d** show cross-sections of a coronary artery; the vessel lumen contains a blood clot attached to the profound neointima by a fibrin network. The vessel wall is further segmented into media and adventitia, the latter containing microvessels. **e** MST-positive control, porcine adrenal 10×. **f** CBS-positive control, porcine adrenal 10×

CBS protein was not detected in the media of any of the CA examined but was localized to the adventitia, in the atheromatous plaques and foam cells of the CA (Figs. 1a and 2). Nitrotyrosine formation (a marker of nitrosative stress/peroxynitrite formation) was only detected in the atheromatous plaque and was 80% more abundant (*p* = 0.056) in the CA of septic animals in contrast to sham (Figs. 1a and 2). No nitrotyrosine staining was found in the native CA. Both sham and septic FBM swine demonstrated strong adipocyte differentiation-related protein (ADRP) expression (*p* = 0.571), while little to no ADRP was found in the CA of the YGP (Fig. 1a, Fig. 3d). Figure 2 shows that CBS and nitrotyrosine immunostaining co-localized in areas of injury, which was also mirrored by ADRP expression (Fig. 3). We were unable to detect any 3-MST expression in the CA samples (Fig. 3c).

## Discussion

This study investigated the topographical expression of the three known endogenous H_2_S-producing enzymes in native and atherosclerotic porcine coronary arteries with and without sepsis. Our main findings are that (i) CSE expression in porcine CA decreases in atherosclerotic pigs and that (ii) this decrease becomes even more pronounced after sepsis.

Endogenously produced H_2_S protects cardiac and vascular tissues from ischemic challenges [26]. In this study, the CA from septic swine presented with decreased CSE expression in the media but increased nitrotyrosine formation in the atheromatous plaque. This is consistent with reports that implicate a loss of CSE expression in vascular smooth muscle cells to be associated with hyperplasia, increased neointima formation, and redox imbalance [27–29]. Our findings further support the view that reduced CSE expression is associated with increased oxidative and nitrosative stress, which may be attributed to either reduced H_2_S and/or diminished nitric oxide bioavailability: nitrotyrosine is an established marker for assessing the nitration of tyrosine residues by peroxynitrite, which is a product of the reaction of NO and superoxide and, thus, a well-established measure of increased oxidative and nitrosative stress [30, 31]. The enhanced oxidative stress along with a reduction in CSE expression might also be due to a reduction in cysteine levels. Cysteine is a product of CSE and a rate-limiting substrate for the synthesis of glutathione, which is an important antioxidant, and its reduction was reported to mirror the severity of sepsis and to be a potential prognosticator of death [32, 33].

Interestingly, CBS was only detected in the atherosclerotic plaques of the animals with CAD, in regions that also displayed abundant nitrotyrosine formation; surprisingly, the CBS expression in the adventitia was not associated with nitrotyrosine formation. Previously, we and others reported that (in contrast to rodents) both human and porcine expressions of CBS are barely detectable in the native kidney [3, 17] and that CBS protein can only be found in small amounts in areas associated with necrosis and injury [3]. These findings are consistent with the congruence of immunohistochemical expression patterns for CBS and ADRP in the CA (Fig. 3) of the co-morbid pigs. ADRP-positive lipid droplets are a common feature of lipid storage diseases and, in particular, in lipid-laden macrophages and increased foam cell formation in atherosclerosis [34], which were found present in both the septic and sham arms.

The family of gasotransmitters includes the physiologically relevant H_2_S, NO, and CO all being known to be endogenously produced, freely penetrate cellular membranes, and share a variety of biological functions [35]. Importantly, there are many examples where these three mediators affect or modulate one another’s action. For instance, H_2_S was shown to upregulate HO-1, an endogenous producer of carbon monoxide (CO), in various settings including in human kidney cells [36] and rodent myocardium [37, 38]. Interestingly, in our experiment, the expression pattern of HO-1 resembled the pattern of CSE expression: it is localized to the media of the CA, was downregulated in CAD, and further significantly suppressed in sepsis. Our finding that the basal levels of HO-1 were decreased with CAD and sepsis is in line with the reports that chronic oxidative stress due to obesity leads to impaired HO-1 production [39, 40]. This is further supported by the increased nitrotyrosine formation in both the CAD and the septic arms which has been shown to impair HO-1 function [41].

The vasodilatory effect of H_2_S depends on its ability to stimulate eNOS activity, as well as to promote cGMP-dependent downstream signaling [42–46]. In our study, the most profound loss of CSE was not only accompanied by lower HO-1 expression but also by a downregulation of eNOS as evidenced in the atherosclerosis and sepsis arm. Although eNOS is an important player in regulating vascular function with anti-atherogenic properties [47, 48], reports on its role in hypercholesterolemia are mixed: Hypercholesterolemia was reported to upregulate eNOS expression in the rabbit aorta [49], while it may be functionally compromised and contribute to endothelial dysfunction in CAD patients [50]. However, in a recent study examining eNOS expression in coronary arteries of patients with/out coronary artery stenosis, Abolhalaj et al. did not find a difference [51]: this is reflective of our finding with regard to the native and CAD arms. Interestingly, the combination of sepsis and CAD demonstrated a profound decrease of eNOS expression, which may be due to the sampling since Lange et al. showed that eNOS is upregulated early in sepsis and then steadily decreases over time [52]. In septic shock, the majority of vascular NO is produced from iNOS; thus, markedly enhanced availability of NO from iNOS might account for the reduction in eNOS expression in CA endothelium in septic animals [53]. The decreased eNOS expression appears to be concomitant with the attenuated expression found for CSE and HO-1. The simultaneous reduction of the enzymes involved in the production of all three gasotransmitters in the CAD animals subjected to sepsis suggests that not only is the vital beneficial/vasculo-protective effect of each of the three mediators (NO, CO, and H_2_S) lost but also their synergistic crosstalk (crucial to vascular function) becomes compromised.

It is worthy of note that, with regard to the H_2_S-producing enzymes, only CSE was found to be locally expressed in the CA. The presence of CBS was found to be localized in the luminal region of the CA, in the blood and clots associated with the atheromatous plaques. There was no MST detectable in any of the CA examined, and it was only found to be positively expressed in coagulated blood, thus suggesting that in this species, MST may be predominantly blood-borne. Confirming previous data in rodents and pigs by Chai et al. and Hedegaard et al. [6, 7], CSE appears to be the most important of the endogenous H_2_S-producing enzymes in the CA. Here, we show for the first time that CSE is localized in the media in vascular smooth muscle cells in CA.

There is a paucity of data on the effects of age on CSE expression. Our study per se does not address this issue, but since the YGP were much younger than the FBM pigs, the apparent loss of CSE expression in the CA may also be due to aging rather than atherosclerosis, although the available data suggest that CSE protein is increased [54] or mRNA expression remains unchanged with increasing age [54, 55]. Thus, it would seem unlikely that age resulted in decreased CSE expression in our model.

The data on YGP, though of a different strain, give a basic idea of the H_2_S-producing enzyme expression in a naïve uninjured animal. In a previously published study, we could not find a difference in CSE protein expression in pre-injury kidney biopsies between FBM and YGP [3]; thus, we were surprised to see this apparent difference between the same two strains in the coronary artery. This suggests that the differences we have now observed in the CA may indeed be due to the atherosclerosis. This is supported by the evidence that atherogenic diets, though not extensively studied, all demonstrate a modulation of the endogenous H_2_S system: reduced H_2_S levels, increased CSE mRNA, and the ameliorated plaque and foam cell formation through the pharmacological administration of an H_2_S donor [2, 56], whereas a restricted caloric diet led to increased H_2_S production [54].

Decreased CSE and HO-1, as well as diminished H_2_S and reduced NO, are implicated in the pathophysiology of atherosclerosis [2, 13, 57–60]. As was reported by Raper & Sibbald, patients suffering from CAD present with a lower cardiac output in response to sepsis than otherwise healthy patients [14]. This is similar to our current findings where, despite the comparable norepinephrine infusion rates (FBM 1.23 (0.6; 3.26) vs YGP 0.61 (0.33; 0.72) μg/(kg min)) [54], the FBM septic swine with atherosclerotic co-morbidity had a lower cardiac output, 87 (62; 130) ml/(kg min), than in our previous study in septic YGP, 131 (117;183) ml/(kg min), using exactly the same experimental design [61], thus suggesting that the FBM swine with atherosclerosis may be a reasonable model to study the effects of CAD in circulatory shock.

### Limitations

Due to the post hoc nature of this study, we were limited to the material available and therefore methodologically relied mostly on immunohistochemistry, although the elucidation of the specific topographical expression of the three known endogenous H_2_S-producing enzymes in native and atherosclerotic porcine coronary arteries with and without sepsis would not be possible with techniques that require tissue homogenization. The localized expression in intact tissue is of relevance due to the ambivalent descriptions in the current literature which may be compromised by contamination with occult blood and its products, i.e., blood clots; “blood cells are known to contain abundant MST activity” that is almost impossible to remove and/or control for in homogenized samples [62]. Finally, our results only draw conclusions based on the expression of the enzymes; for the aforementioned reasons, we were unable to determine activity and/or substrate availability.

## Conclusions

Taken together, we conclude that CSE in a clinically relevant resuscitated porcine model of sepsis appears to be the only one of the endogenous H_2_S enzymes to be locally produced and directly affected by both atherosclerosis and concomitant sepsis. We speculate that the reduction of CSE, concomitant with reduced HO-1 and eNOS, and coinciding with enhanced oxidative/nitrosative stress may contribute to the development of coronary dysfunction in sepsis. Further studies, aimed at replacing H_2_S (or simultaneously replacing H_2_S, NO, and CO) in septic pigs are needed to directly test the potential beneficial effect of gasotransmitter donation on the cardiac function in sepsis.

## Abbreviations

3-MST: 3-Mercaptopyruvate sulfurtransferase
ADRP: Adipocyte differentiation-related protein
CA: Coronary artery
CAD: Coronary artery disease
CBS: Cystathionine-β-synthase
CO: Carbon monoxide
CSE: Cystathionine-γ-lyase
CVP: Central venous pressure
eNOS: Endothelial nitric oxide synthase
FBM: Familial hypercholesterolemia Bretoncelles Meishan
H_2_S: Hydrogen sulfide
HO-1: Heme oxygenase-1
IQR: Interquartile range
LDL: Low-density lipoprotein
MAP: Mean arterial pressure
NO: Nitric oxide
YGP: Young German pig
